# Tailoring Mechanical Properties of Al-Cr-Cu-Fe-Mn-Ni Complex Concentrated Alloys Prepared Using Pressureless Sintering

**DOI:** 10.3390/ma18174068

**Published:** 2025-08-30

**Authors:** Tiago Silva, Augusto Lopes

**Affiliations:** Department of Materials and Ceramic Engineering, CICECO-Aveiro Institute of Materials, University of Aveiro, 3810-193 Aveiro, Portugal; augusto@ua.pt

**Keywords:** complex concentrated alloys, mechanical properties, powder metallurgy, pressureless sintering, microstructure, thermodynamic calculations

## Abstract

Complex concentrated alloys (CCAs) have attracted significant attention due to their potential to develop materials with enhanced properties, such as increased hardness and strength. These properties are strongly influenced by the chemical composition and the processing method used. Body-centred cubic (BCC) structures are known to have high hardness but low fracture toughness, whereas face-centred cubic (FCC) structures typically exhibit lower hardness but higher toughness. In this study, Al-Cr-Cu-Fe-Mn-Ni CCAs with three distinct compositions were produced using pressureless sintering. One set of samples was prepared with equiatomic composition (composition E), whereas the compositions of the other two sets were defined based on thermodynamic calculations to obtain sintered samples predominantly formed by BCC (composition B) or FCC (composition F) phases. The samples were characterized using X-ray diffraction, scanning and transmission electron microscopy, energy-dispersive X-ray spectroscopy, electron backscatter diffraction, density measurements, hardness measurements, and uniaxial compression tests. For all compositions, good agreement was obtained between the phases predicted by thermodynamic calculations and those experimentally detected. In addition, significant differences in the mechanical properties were observed between samples with each composition. The samples with composition B exhibited the highest hardness, but almost no ductility. In contrast, samples with composition F showed the lowest yield strength and hardness, but the highest ductility. Samples with composition E had intermediate values between those of samples B and F. These differences were attributed to differences in the proportions and properties of the BCC and FCC phases in each composition and demonstrate that the mechanical properties of Al-Cr-Cu-Fe-Mn-Ni CCAs can be tailored using compositions defined based on thermodynamic calculations.

## 1. Introduction

Recent research has increasingly focused on metallic materials composed of more than two elements in near-equiatomic proportions [1,2,3]. The distinctive feature of these alloys, compared with conventional ones, is the presence of multiple principal elements [1,2,3]. Known as complex concentrated alloys (CCAs), these materials offer new opportunities for developing materials with enhanced properties, such as increased hardness, improved wear resistance, reduced thermal conductivity, enhanced corrosion resistance, and superior strength at both ambient and elevated temperatures [1,2,3,4].

CCAs are defined as alloys composed of more than two elements, each with atomic concentrations ranging from 5% to 35% [2,3,5]. This category includes high-entropy alloys and can exhibit various phases, such as ordered and disordered solid solutions (SSs), intermetallic compounds (IMs), or a combination of both [2,5]. The SSs in these alloys are typically classified based on their atomic structure as face-centered cubic (FCC), body-centered cubic (BCC), or hexagonal close-packed (HCP) [2,5]. In most CCAs, multiple phases coexist. For example, in Al-Cr-Cu-Fe-Mn-Ni CCAs, the presence of two BCC phases (one ordered and one disordered), as well as one FCC phase, has been reported [6,7,8,9,10,11,12]. As a result, these alloys exhibit a remarkable set of properties, including a hardness of approximately 500 HV, relatively low density, resistance to softening during annealing, excellent oxidation resistance, and superior corrosion resistance compared to 304 L stainless steel [6,7]. The processing route also plays a fundamental role in CCA properties [2]. Arc melting [13,14,15] and powder metallurgy [15,16,17] are the main manufacturing methods for producing these alloys. The latter offers several advantages, including low cost and homogeneity of the final products, can be easily adapted for large-scale production, and is suitable for systems with components with a substantial range of melting temperatures [16,17]. Powder metallurgy typically involves mechanical alloying or milling, followed by compaction and sintering [2,10,11]. Sintering of CCA powders is predominantly carried out using non-conventional techniques such as spark plasma sintering (SPS) [1,2,18,19]. However, this method is generally limited to small samples with simple geometries [18,19]. In contrast, pressureless sintering allows for the consolidation of components with different dimensions and shapes and requires relatively simple equipment. Nonetheless, it presents challenges such as reduced densification rates and, typically, higher porosity of the final products, which can negatively affect their properties [16,20]. In the case of AlCrCuFeMnNi CCAs produced via pressureless sintering, the formation of a liquid phase during the process has been observed to promote the coalescence of oxide particles, decreasing the hardness of the samples. This effect, however, can be mitigated by the addition of Mg, which promotes a more uniform and finer distribution of oxide particles, thereby leading to increased hardness [8].

In the last few years, thermodynamic calculations were employed in several studies to predict phase evolution in multicomponent systems and to identify compositions that generate phases that promote the desired properties for specific applications [2,21,22,23,24,25,26]. However, this approach has been applied to a very limited set of alloy compositions, which does not include Al-Cr-Cu-Fe-Mn-Ni CCAs.

Therefore, the primary objective of this work is to employ thermodynamic calculations to define the composition that allow the production of Al-Cr-Cu-Fe-Mn-Ni CCAs predominantly formed by BCC or FCC phases and to compare their properties with those of samples with equiatomic composition prepared under similar conditions by pressureless sintering.

## 2. Materials and Methods

### 2.1. Thermodynamic Calculations

The composition of the samples predominantly formed by FCC (composition F) or BCC (composition B) phases was defined based on thermodynamic calculations performed using the Opticalc module of the FactSage 7.3 software (GTT-Technologies Aachen, Germany), combined with the SGTE 2017 database [27]. This module uses the mesh adaptive direct search (MADS) algorithm to automatically search for optimal conditions (compositions, temperatures, and activities) for a defined set of constraints [24,25,26]. In this work, it was imposed that the molar concentration of each element be between 5% and 35%, as defined for CCAs. Thermodynamic calculations were also performed to predict the evolution of the phases in equilibrium at different temperatures for compositions B and F and for the equiatomic composition (composition E).

### 2.2. Sample Preparation

Samples with compositions F, B, and E were prepared from commercial powders of Al, Cr, Cu, Fe, Mn, and Ni with purities higher than 99.5% and particle size between 12 μm and 350 μm (Al, Fe, and Ni with less than 45 μm and Cr, Cu, and Mn with less than 350 μm, all provided by abcr, Karlsruhe, Germany). For each of the three compositions (F, B, and E), 1 at. % of Mg was added to reduce oxides coalescence during sintering [8,9].

The powders were weighed in the respective proportions and wet-milled for 15 h in a 90% helium and 10% hydrogen atmosphere. This process was carried out using a planetary mill (Retsch PM100, Haan, Germany) operating at 250 rpm, with steel vials and balls as the milling media, toluene as the process control agent, and a ball-to-powder ratio of 10:1. After being vacuum dried at room temperature, the powder was used to prepare, by uniaxial pressing followed by isostatic pressing, cylindrical samples for compression tests and parallelepiped samples for Young’s modulus measurements. A pressure of 150 MPa was used for uniaxial pressing (Craver Press, High Wycombe, UK) followed by isostatic pressing at 200 MPa (Autoclave Engineers, Erie, PA, USA). The samples were then sintered in a tubular furnace at temperatures defined based on the thermodynamic calculations (1050 °C for composition E and 1150 °C for compositions B and F) for 1 h in an atmosphere of 90 vol. % helium and 10 vol. % hydrogen.

The powders and sintered samples were characterized using X-ray diffraction (XRD) with a PANalytical X’Pert Pro diffractometer (Malvern, UK) and Cu-Kα radiation, scanning electron microscopy (SEM, Hitachi SU-70, Tokyo, Japan), transmission electron microscopy (TEM, JEOL 2200FS, Tokyo, Japan), and energy-dispersive X-ray spectroscopy (EDS, Bruker Quantax, Billerica, MA, USA and Oxford Ultim Max systems, Oxfordshire, UK). Particle size distributions before and after milling were analyzed using a laser scattering technique (Horiba LA-960, Kyoto, Japan). The sintered samples were polished (using diamond paste from 15 to 1 µm and colloidal alumina of 0.05 µm) and characterized using electron backscattered diffraction (EBSD, Bruker Quantax Cryst Align, Billerica, MA, USA) to evaluate the phase distribution using information from PDF cards of the phases identified with XRD. For TEM analysis, the samples were mechanically ground to a thickness of 20 μm and ion-beam polished to create a hole (Gatan 691 Precision Ion Polishing System, Pleasanton, CA, USA) at 1.5 kV to 4 kV and an incidence angle between 4° and 2°. Mechanical properties were evaluated with uniaxial compression tests using cylindrical samples with 13 mm of diameter and 25 mm of height and a constant rate of 1 mm/min (Shimadzu AG-25TA, Kyoto, Japan, equipped with 250 kN load cell) and Vickers hardness tests with a 19.6 N (2 kgf) load (Wilson VH1102, Lake Bluff, IL, USA). The Young’s modulus values were calculated using the fundamental resonant frequencies measured according to ASTM E 1876-01 standard in parallelepiped samples with dimensions of 50 mm × 5 mm × 5 mm. The density of the samples was measured using the weight and dimensions (before sintering) and the Archimedes principle with water (after sintering).

## 3. Results and Discussion

### 3.1. Thermodynamic Calculations

Table 1 presents the proportions of elements needed to achieve the equiatomic composition and, based on thermodynamic calculations, the maximum amount of FCC or BCC phase at room temperature in a system containing Al, Cr, Cu, Fe, Mn, and Ni. As can be seen, the main differences between the F and B compositions are in the higher amounts of Al and Ni and the lower proportions of Cu, Fe, and Mn in the latter formulation. This agrees with previous studies that showed that the amount of BCC phase in CCAs is favoured by higher Al content, while increasing the relative amounts of Cu and Mn promotes the formation of the FCC phase [2,5]. The presence of Ni also favours the formation of the FCC phases but tends to form intermetallic phases with Al contributing to an increase in the proportion of the BCC phases [2,5,12].

**Figure 1:**
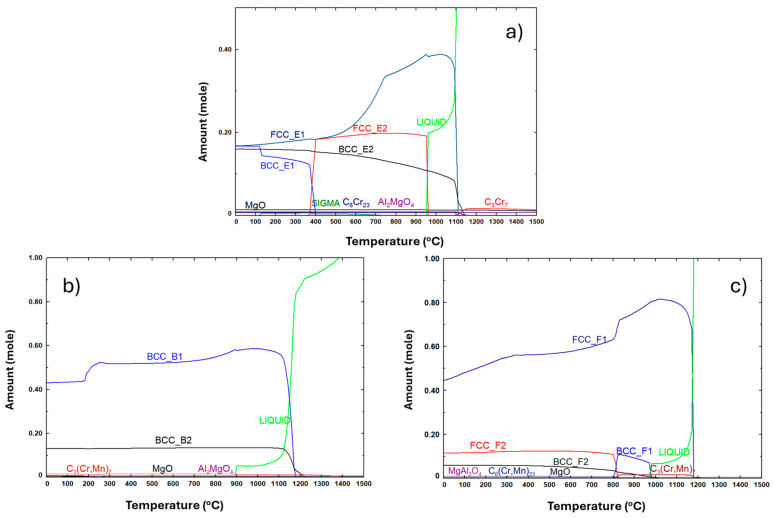
Relative amounts of phases at equilibrium predicted by thermodynamic calculations at different temperatures for (**a**) composition E, (**b**) composition B, and (**c**) composition F.

Figure 1 shows the phases at equilibrium at different temperatures predicted by thermodynamic calculations for the three compositions with the addition of 1% of Mg. In these calculations, the presence of 2% of O and 5% of C was also assumed, detected using EDS in the sintered samples. These latter chemical elements were also identified in the initial powders, and some of the carbon content may have originated from the milling process.

At room temperature, the predicted phases for composition E include an FCC_E1 phase rich in Cu and Al, a BCC_E1 phase rich in Fe, Mn, Al, and Cr, a BCC_E2 phase rich in Al and Ni, a Sigma phase rich Cr, Mn, and Fe, C_6_Cr_23_, MgO, and Al_2_MgO_4_. These results are similar to those reported in a previous study on the microstructural characterization of equiatomic samples prepared from powders with composition different from that used in this work [8]. For composition B, a BCC_B1 phase rich in Al, Fe, and Mn, a BCC_B2 phase rich in Al and Ni, C_3_(Cr,Mn)_7_, MgO, and Al_2_MgO_4_ are predicted. For composition F, it is predicted to have an FCC_F1 phase rich in Fe and Mn, a FCC_F2 phase rich in Cu, a BCC_F2 phase rich in Al and Ni, C_6_(Cr,Mn)_23_, MgO, and Al_2_MgO_4_. Regarding the relative amounts of BCC and FCC phases, these results suggest that, at room temperature, composition B mainly consists of BCC phases, as intended, with a small fraction of carbides and oxides. In contrast, the composition F should be formed mostly by FCC phases, with a small fraction of BCC phase, carbides, and oxides. Composition E corresponds to an intermediate situation, presenting both BCC and FCC phases in an approximate ratio of 2:1 and small proportions of carbides and oxides.

Another important result from these thermodynamic calculations is the melting temperature. For composition B, the liquid phase is predicted to begin forming at approximately 900 °C. For the F and E compositions, this occurs at approximately 970 °C and 950 °C, respectively. However, compositions B and F present similar amounts of liquid phase at 1150 °C as composition E at 1050 °C, which justifies the use of these temperatures for sintering the samples.

### 3.2. Powder Characterization

**Figure 2:**
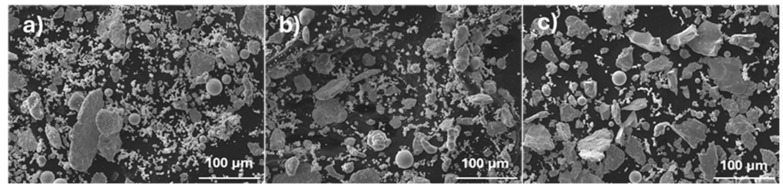
SEM images of powder before milling used to produce (**a**) E, (**b**) B, and (**c**) F samples.

Figure 2 shows the mixtures of the initial powders used to prepare samples with the investigated compositions. All are composed of particles of different sizes and shapes. EDS analysis showed that the smaller, irregular particles consist mainly of Fe, Ni, or Al. On the other hand, Cu or Mg and Cr or Mn were detected primarily in the spherical and plate-like particles, respectively. For all the mixtures, the measured average chemical composition closely matched the nominal one.

**Figure 3:**
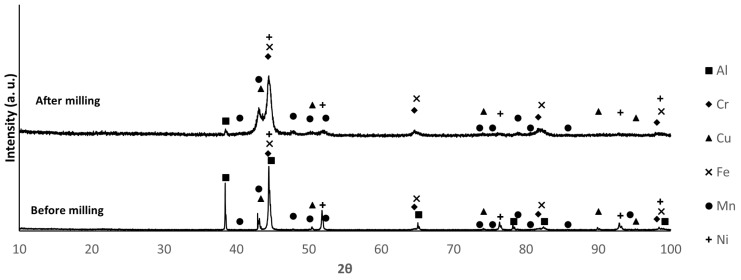
XRD patterns of sample E before and after milling. (PDF card index: Al (04-012-3402); Cr: (04-003-2918); Cu: (01-070-3039); Fe: (04-002-8852); Mn (04-007-1944); Ni: (04-004-6807) [28]).

**Figure 4:**
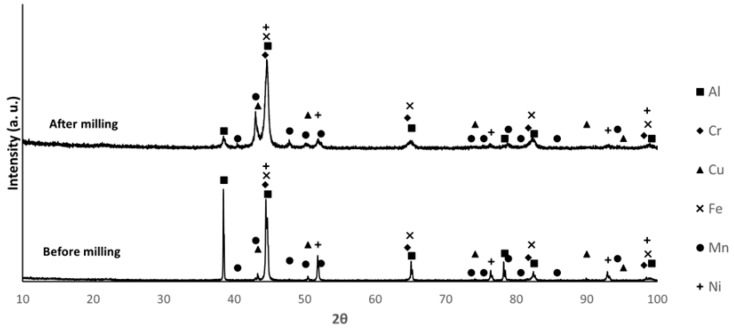
XRD patterns of sample B before and after milling. (PDF card index: Al (04-012-3402); Cr: (04-003-2918); Cu: (01-070-3039); Fe: (04-002-8852); Mn (04-007-1944); Ni: (04-004-6807) [28]).

**Figure 5:**
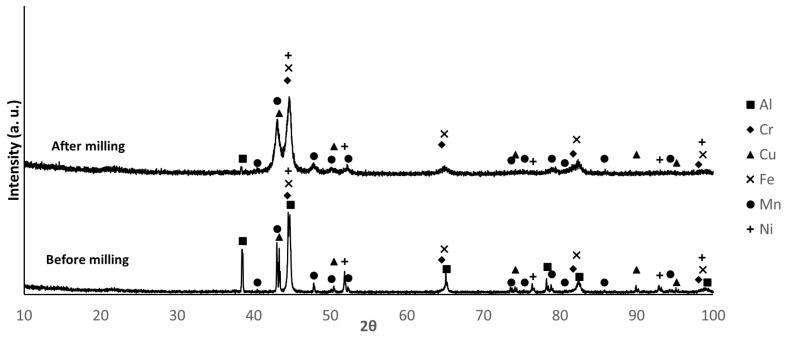
XRD patterns of sample F before and after milling. (PDF card index: Al (04-012-3402); Cr: (04-003-2918); Cu: (01-070-3039); Fe: (04-002-8852); Mn (04-007-1944); Ni: (04-004-6807) [28]).

The X-ray diffractograms of the mixtures before and after milling are shown in Figure 3, Figure 4 and Figure 5. The identified phases are consistent with the presence of Al, Cr, Cu, Fe, Mn, and Ni. The main difference between the diffraction patterns is the broadening of the peaks, attributed to the decrease in crystallite size and the increase in lattice strain induced by the milling process. For composition B, the increased intensity of the second and third diffraction maxima after milling, compared to their corresponding values before milling, may indicate that a new phase was formed during this processing step. The non-detection of Mg diffraction peaks is justified by the low relative amount of this element in the mixtures.

By comparing the relative intensities of the diffraction maxima for the different compositions, the largest differences are observed for the Al, Cu, and Mn phases. In composition B, the Al peaks show higher intensity, while the Cu peaks show lower intensity, as expected, considering the proportions of these elements in the mixture. For composition F, the Mn and Al peaks present higher and lower intensity, respectively. The intensity of the Al peaks in sample E falls between those of samples B and F. These results are consistent with the proportions presented in Table 1.

**Figure 6:**
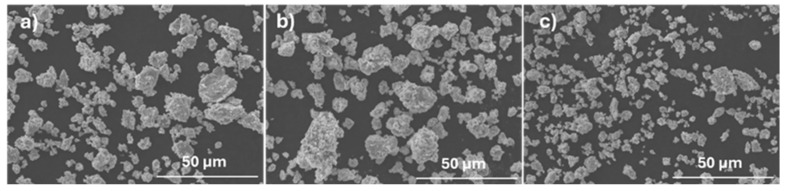
SEM images of powder after milling used to produce (**a**) E, (**b**) B, and (**c**) F samples.

**Figure 7:**
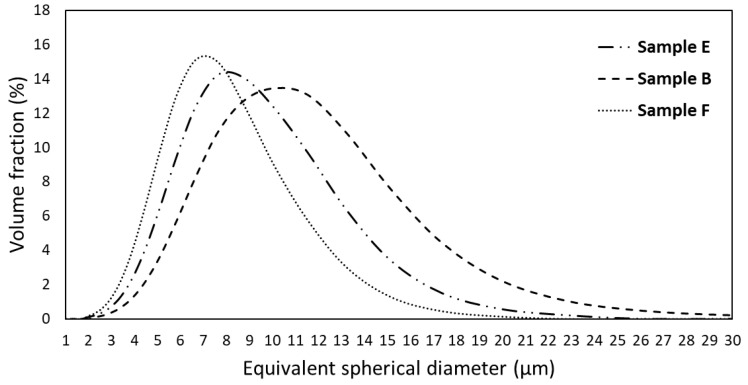
Particle size distribution of the powders after milling.

**Figure 8:**
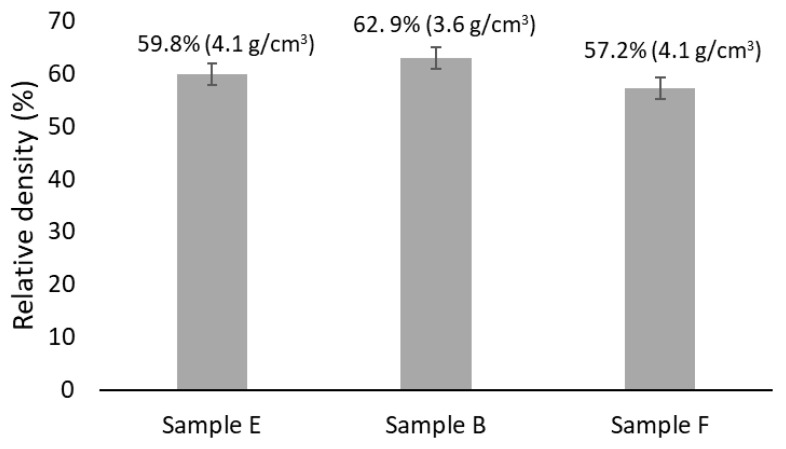
Relative density of the samples after uniaxial and isostatic cold pressing. The density values are presented in brackets.

After milling, the SEM images (Figure 6) and EDS analyses showed that the obtained powders predominantly consist of agglomerates with a high degree of chemical homogeneity. The particle size distribution curves (Figure 7) indicate that the powders exhibit a unimodal distribution with average sizes of 6.7 μm, 7.5 μm, and 9.3 μm for powders with F, E, and B compositions, respectively. The maximum particle sizes are approximately 20 μm (for F composition), 25 μm (for E composition) and 30 μm (for B composition). Despite these differences, the degree of compaction of the powders after uniaxial pressing was not substantially affected (Figure 8), resulting in samples with average relative densities between 57% and 63%.

### 3.3. Sample Characterization After Sintering

**Figure 9:**
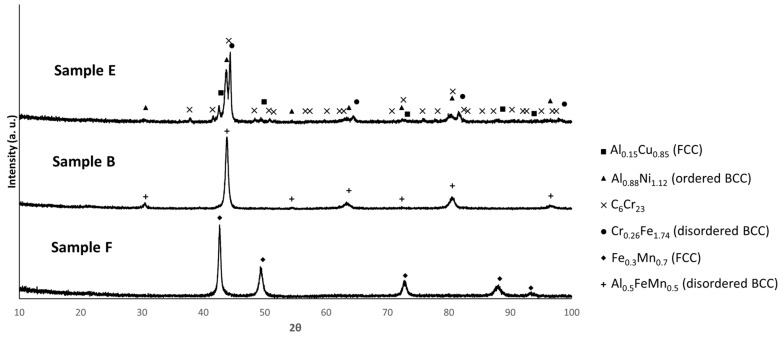
XRD patterns of sintered samples E, B, and F. (PDF card index: Al_0.15_Cu_0.85_ (01-077-6740); Al_0.88_Ni_1.12_ (04-002-1233); Cr_23_C_6_ (04-004-3124); Cr_0.26_Fe_1.74_ (00-034-0396); Fe_0.3_Mn_0.7_ (01-071-8284), Al_0.5_FeMn_0.5_ (04-022-6249) [28]).

Figure 9 shows the diffractograms of the samples after sintering. For sample E, the pattern is consistent with the presence of Al_0.88_Ni_1.12_ (ordered BCC), Cr_0.26_Fe_1.74_ (disordered BCC), Al_05.1_Cu_0.85_ (FCC) and C_6_Cr_23_. In contrast, the patterns of samples B and F are both consistent with the presence of a single phase, namely Al_0.5_FeMn_0.5_ (disordered BCC) for sample B and Fe_0.3_Mn_0.7_ (FCC) for sample F. For all the compositions, the identified phases were predicted by the thermodynamic calculations. However, the Sigma phase (for composition E), BCC_B2 (for composition B), BCC_F2 and FCC_F2 (for composition F), the carbides C_3_(Cr,Mn)_7_ (for composition B) and C_6_(Cr,Mn)_22_ (for composition F), and the oxides MgO and Al_2_MgO_4_ (for all compositions) predicted by these calculations were not detected by XRD, probably due to their low amounts.

**Figure 10:**
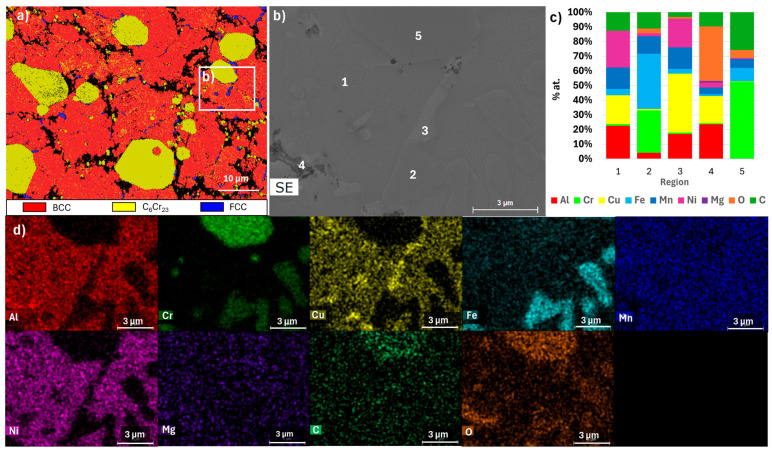
Results obtained for sample E. (**a**) EBSD phase map of BCC, C_6_Cr_23_, and FCC; (**b**) SEM image of the area defined in (**a**); (**c**) results of quantitative analyses by EDS at the regions identified in the SEM image; (**d**) distribution maps (images below) of Al, Cr, Cu, Fe, Mn, Ni, Mg, C, and O.

**Figure 11:**
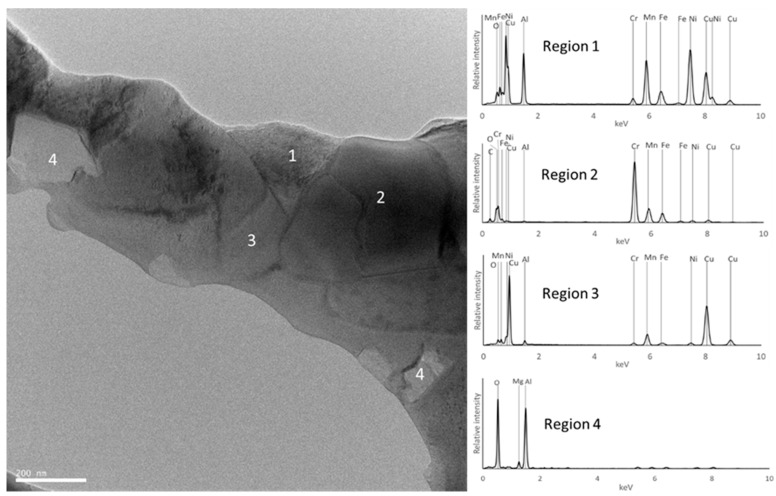
TEM image of sample E and EDS spectra from the regions identified with numbers.

**Figure 12:**
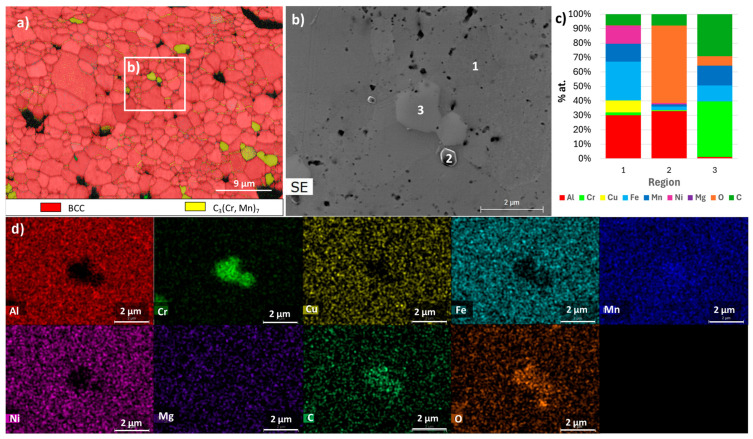
Results obtained for sample B. (**a**) EBSD phase map of BCC and C_3_(Cr, Mn)_7_; (**b**) SEM image of the area defined in (**a**); (**c**) results of quantitative analyses by EDS at the regions identified in the SEM image; (**d**) distribution maps (images below) of Al, Cr, Cu, Fe, Mn, Ni, Mg, C, and O.

**Figure 13:**
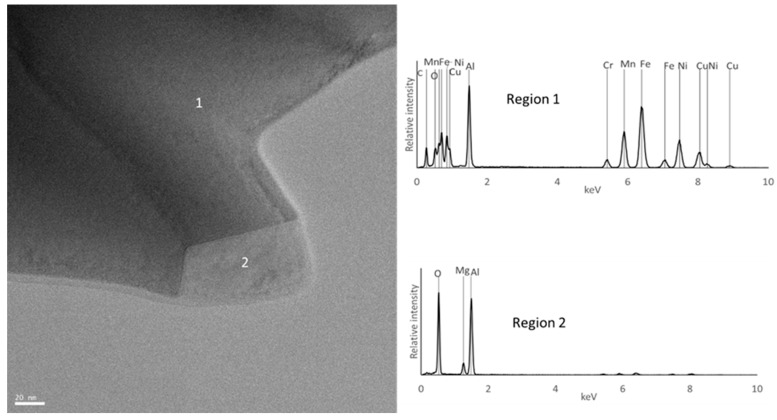
TEM image of sample B and EDS spectra from the regions identified with numbers.

**Figure 14:**
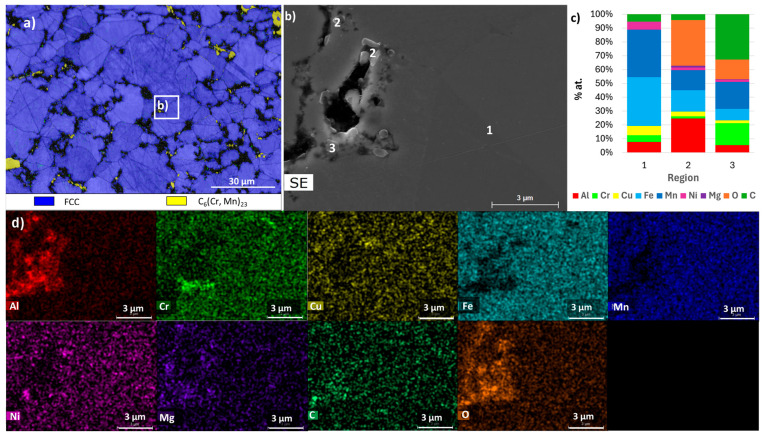
Results obtained for sample F. (**a**) EBSD phase map of FCC and C_3_(Cr, Mn)_7_; (**b**) SEM image of the area defined in (**a**); (**c**) results of quantitative analyses by EDS at the regions identified in the SEM image; (**d**) distribution maps (images below) of Al, Cr, Cu, Fe, Mn, Ni, Mg, C, and O.

**Figure 15:**
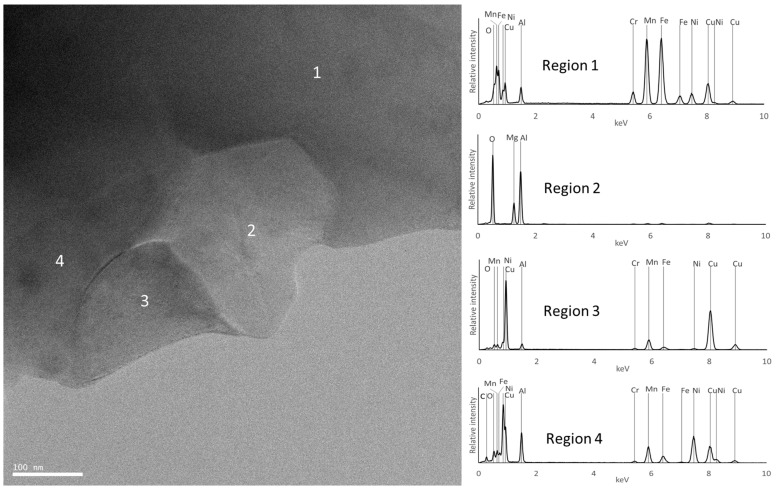
TEM image of sample F and EDS spectra from the regions identified with numbers.

Figure 10, Figure 11, Figure 12, Figure 13, Figure 14 and Figure 15 show the EBSD phase maps, SEM and TEM images and EDS analysis results obtained on the sintered samples.

The EBSD maps confirm the presence of C_6_Cr_23_, FCC, and BCC phases in sample E. Furthermore, they show that sample B exhibits a smaller grain size compared to sample E or F, presenting average values of 2.9 µm, 10.7 µm, and 13.5 µm, respectively. It is important to note that the diffraction patterns of the ordered and disordered BCC phases are very similar, making it difficult to distinguish them by EBSD. However, the presence in sample E of a Cr and Fe-rich phase and an Al and Ni-rich phase, corresponding to disordered and ordered BCC structures, respectively, is confirmed by EDS (regions 2 and 1 in Figure 10 and Figure 11). This technique also confirmed the presence of Al and Cu-rich areas corresponding to an FCC phase (region 3 in Figure 10 and Figure 11) and oxides containing Al and Mg (region 4 in Figure 10 and Figure 11). These oxides were small in size and quantity and showed a relatively uniform distribution, mainly along the grain boundaries.

In the B samples, the presence of a large amount of the BCC (disordered) phase rich in Al, Fe, and Mn was confirmed (region 1 in Figure 12 and Figure 13), carbides rich in Cr, Fe, and Mn (region 3 in Figure 12), and oxides rich in Al and Mg (region 2 in Figure 12 and Figure 13) were also detected.

For the F samples, the analyses confirmed that the samples consist mostly of an FCC phase rich in Fe and Mn (region 1 in Figure 14 and Figure 15), carbides rich in Cr and Mn (region 3 in Figure 14), and oxides rich in Al and Mg (region 2 in Figure 14 and Figure 15). It should be noted that the TEM-EDS analysis also revealed the presence of a Cu-rich phase (region 3 in Figure 15) and an Al- and Ni-rich phase (region 4 in Figure 15), which is compatible with the presence of the FCC_F2 and BCC_F2 phases predicted by the thermodynamic calculations for this composition.

Table 2 summarizes all the phases identified by XRD, EBSD, and EDS, as well as those predicted at room temperature by thermodynamic calculations for the three compositions studied. As can be seen, there is good agreement between the results obtained, with nearly all predicted phases being experimentally confirmed. The exceptions were the sigma phase for composition E, the BCC_B2 phase rich in Al and Ni for composition B, and the MgO phase for all compositions, probably due to their low quantities in the samples.

**Figure 16:**
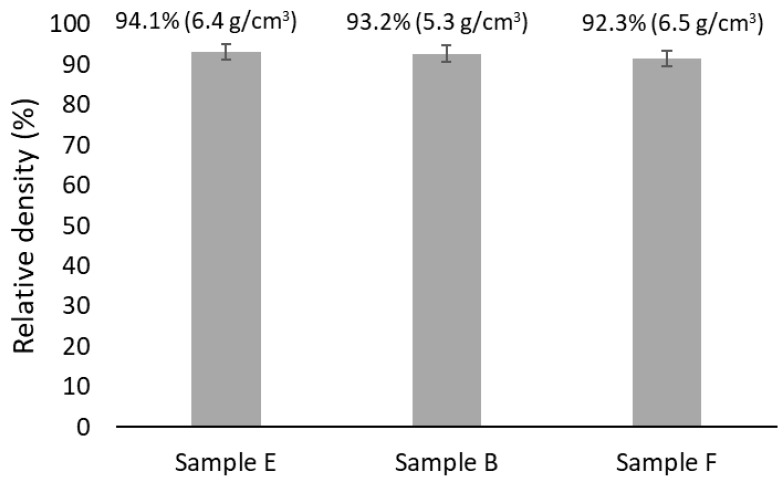
Relative density of the sintered samples. The density values are presented in brackets.

Figure 16 shows, in brackets, the density values of the samples after sintering. The same figure also presents the corresponding relative density values, which were calculated as the ratio of the experimental density to the theoretical density, considering the phase proportions predicted for each composition. As can be seen, the sintered samples exhibit similar relative densities, all exceeding 90%. This is consistent with the low porosity levels observed by SEM and aligns with the presence of a liquid phase in comparable amounts (as predicted by the thermodynamic calculations), which enhances the densification process by facilitating particle rearrangement and diffusion processes [29].

**Figure 17:**
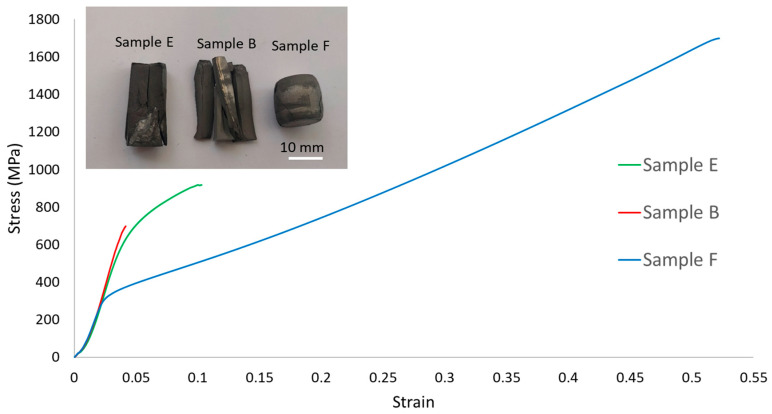
Uniaxial compression stress–strain curves for samples E, B, and F. An image of the samples after the tests is also presented.

Despite exhibiting similar porosity values, the samples with the three compositions showed significant differences in their mechanical properties. As presented in Figure 17, the uniaxial compression stress–strain curves of each sample nearly overlap during the initial stage corresponding to the elastic domain, in which the stress increases with the strain at an almost constant rate. This is in good agreement with the relatively small variations (less than 9%) in the dynamic Young’s modulus values obtained for the different samples (Table 3).

However, after this initial stage, the samples show markedly different behaviours. While the rupture of sample B occurs almost immediately after the elastic domain, for samples E and F, this occurs after approximately 0.05 and 0.43 of plastic strain, respectively. The increase in stress after yielding is a result of work-hardening phenomena and the increase in the cross-sectional area of the samples during the compression test (Figure 17). For sample E, these effects result in a maximum compressive stress close to 900 MPa, while for sample F, this value reaches almost 1700 MPa.

Another significant difference observed in the uniaxial compression stress–strain curves is the yield stress value at 0.2% of strain. For samples B and E, this value is relatively similar (approximately 700 MPa and 650 MPa, respectively) but significantly higher than that of sample F (which is around 300 MPa). This agrees with the Vickers hardness values measured for the samples (Table 3), as an increase in the yield strength corresponds to an increase in the material hardness. In this study, there was an increase of almost 75% in the hardness value of sample B compared to that exhibited by sample F.

In most of the studies conducted on CCAs containing Al, Cr, Cu, Fe, Mn, and Ni, mechanical characterization was carried out through uniaxial tensile or hardness tests on samples obtained by casting [10,11]. To the authors’ knowledge, uniaxial compression tests have not been used. Rao et al. [10] performed uniaxial tensile tests on Al_0.4_Cr_0.4_CuFe_0.4_MnNi and reported a tensile strength and strain values close to 1000 MPa and 0.07, respectively. Nguyen et al. [11] obtained approximately 600 MPa in tensile strength, 0.02 in strain, and a hardness of 518 HV for the AlFeMnNiCrCu_0.5_ CCA. These values are similar to those measured in this work by uniaxial compression tests for samples E, except that the hardness is higher, probably due to the lower porosity of the as-cast samples. However, hardness values between 380 and 402 HV have been reported for equiatomic CCA samples obtained by pressureless with a relative density of approximately 96% [8].

Many studies conducted on CCAs containing Al, Cr, Cu, Fe, Mn, and Ni have shown that the hardness and ductility are significantly influenced by the relative amount of BCC and FCC phases in the tested samples [2,6,10,11,12,13,14]. Samples with high amounts of BCC phases tend to exhibit higher hardness but low ductility, while FCC phases usually promote higher ductility but lower hardness [1,2,11,12,13,15]. This explains the differences in mechanical behaviour observed in this work for the samples with the studied compositions, specifically the highest hardness but lowest deformability of sample B (mainly formed by BCC phases), the lowest hardness but highest deformability of sample F (consisting mostly of FCC phase), and the intermediate values of these properties presented by sample E (with both BCC and FCC phases). Also noteworthy are the relatively small differences between the mechanical behaviour of samples E and B, which is consistent with the higher proportion of BCC than FCC phases in the former. It should be also noted that the smaller grain size of sample B likely contributes to its higher hardness and yield stress [30]. However, this effect is expected to be relatively small and cannot justify the differences observed between E and F samples, whose grain sizes are similar.

These results show that the mechanical properties of Al-Cr-Cu-Fe-Mn-Ni CCAs can be tuned by controlling the relative amounts of the BCC and FCC phases through a proper definition of the alloy composition. Moreover, this study shows that such optimization can be achieved using thermodynamic calculations, thereby eliminating the need for a trial-and-error approach and enabling the rapid development of CCAs with customized characteristics for specific applications.

## 4. Conclusions

In this work, pressureless sintered Al-Cr-Cu-Fe-Mn-Ni CCAs were prepared with equiatomic composition (samples E) and compositions predominantly formed by either BCC (samples B) or FCC (samples F) phases, whose nominal compositions were determined based on thermodynamic calculations.

For all the compositions, good agreement was observed between the phases predicted by the calculations and those experimentally identified using XRD, SEM, TEM, EDS, and EBSD. Despite having similar relative densities (between 92% and 94%), the samples with the three studied compositions showed significant differences in their mechanical behaviour during uniaxial compression and hardness tests, specifically:

- Samples B showed the highest yield stress (approximately 700 MPa) and hardness (367 HV2) values but almost no ductility, failing shortly after the elastic domain.
- In contrast, samples F exhibited the lowest yield stress (approximately 300 MPa) and hardness (211 HV2) values but the highest ductility, reaching a plastic strain of around 0.43.
- Samples E, containing both BCC and FCC phases, showed yield stress (650 MPa), hardness (357 HV2) and maximum plastic strain (0.05) values between those exhibited by samples B and F.

These differences were attributed to the higher hardness but lower ductility of the BCC phases, the higher ductility but lower hardness of the FCC phases, and the different proportions of these phases in each sample.

In a broader context, this study demonstrates that the mechanical properties of Al-Cr-Cu-Fe-Mn-Ni CCAs can be optimized using compositions defined based on thermodynamic calculations.

## Figures and Tables

**Table 1:** Proportions (%) of Al, Cr, Cu, Fe, Mn, and Ni used in the studied compositions.

Composition	Al	Cr	Cu	Fe	Mn	Ni
E	16.6	16.6	16.6	16.6	16.6	16.6
B	35.0	5.0	7.5	25.0	15.0	12.5
F	10.6	5.0	10.7	32.9	35.0	5.8

**Table 2:** Phases predicted by thermodynamic calculations and experimentaly detected by XRD, EBSD, SEM-EDS and TEM-EDS.

Composition	Phases Predicted by Thermodynamic Calculations (at 25 °C)	Phases Identified by			
		XRD	EBSD	SEM-EDS	TEM-EDS
E	FCC_E1 rich in Cu and Al	Al_0.15_ Cu_0.85_ (FCC)	FCC	Region 3	Region 3
	BCC_E1 rich in Fe, Mn, Al and Cr	Cr_0.26_Fe_1.74_ (disordered BCC)	BCC	Region 2	Region 2
	BCC_E2 rich in Al and Ni	Al_0.88_Ni_1.12_ (ordered BCC)	BCC	Region 1	Region 1
	C_6_Cr_23_	C_6_Cr_23_	C_6_Cr_23_	Region 5	Not detected
	Sigma	Not detected	Not detected	Not detected	Not detected
	MgO	Not detected	Not detected	Not detected	Not detected
	Al_2_MgO_4_	Not detected	Not detected	Consistent with composition of region 4	Region 4
B	BCC_B1 rich in Al, Fe and Mn	Al_0.5_FeMn_0.5_ (disordered BCC)	BCC	Region 1	Region 1
	BCC_B2 rich in Al and Ni	Not detected	BCC	Not detected	Not detected
	C_3_(Cr,Mn)_7_	Not detected	C_3_(Cr,Mn)_7_	Consistent with composition of region 3	Not detected
	MgO	Not detected	Not detected	Not detected	Not detected
	Al_2_MgO_4_	Not detected	Not detected	Consistent with composition of region 2	Region 2
F	FCC_F1 rich in Fe and Mn	Fe_0.3_Mn_0.7_ (FCC)	FCC	Region 1	Region 1
	FCC_F2 rich in Cu	Not detected	FCC	Not detected	Region 3
	BCC_F2 rich in Al and Ni	Not detected	Not detected	Not detected	Region 4
	C_6_(Cr,Mn)_23_	Not detected	C_3_(Cr,Mn)_7_	Consistent with composition of region 3	Not detected
	MgO	Not detected	Not detected	Not detected	Not detected
	Al_2_MgO_4_	Not detected	Not detected	Consistent with composition of region 2	Region 2

**Table 3:** Dynamic Young’s modulus and Vickers hardness values of samples E, B, and F.

Samples	Dynamic Young’s Modulus (GPa)	Vickers Hardness (HV2)
E	145 ± 1	357 ± 15
B	135 ± 4	367 ± 6
F	147 ± 4	211 ± 10

## Data Availability

The raw data supporting the conclusions of this article will be made available by the authors on request.

## Abbreviations

The following abbreviations are used in this manuscript:

CCAs	Complex Concentrated alloys
BCC	Body-centred cubic
FCC	Face-centred cubic
MADS	Mesh Adaptive Direct Search
XRD	X-ray diffraction
SEM	Scanning Electron Microscopy
TEM	Transmission Electron Microscopy
EBSD	Electron Backscatter Diffraction
EDS	Energy Dispersive Spectroscopy
